# Direct evidence of catalyst reduction on dye and catalyst co-sensitized NiO photocathodes by mid-infrared transient absorption spectroscopy†
†Electronic supplementary information (ESI) available. See DOI: 10.1039/c8sc00990b


**DOI:** 10.1039/c8sc00990b

**Published:** 2018-05-08

**Authors:** M. Gilbert Gatty, S. Pullen, E. Sheibani, H. Tian, S. Ott, L. Hammarström

**Affiliations:** a Physical Chemistry , Department of Chemistry , Ångström Laboratory , Uppsala University , Box 523 , 75120 Uppsala , Sweden . Email: leif.hammarstrom@kemi.uu.se; b Organic Chemistry , Department of Chemistry , Chemical Science and Engineering , KTH , Royal Institute of Technology , Teknikringen 30 , 100 44 Stockholm , Sweden

## Abstract

Co-sensitization of molecular dyes and catalysts on semiconductor surfaces is a promising strategy to build photoelectrodes for solar fuel production. In such a photoelectrode, understanding the charge transfer reactions between the molecular dye, catalyst and semiconductor material is key to guide further improvement of their photocatalytic performance. Herein, femtosecond mid-infrared transient absorption spectroscopy is used, for the first time, to probe charge transfer reactions leading to catalyst reduction on co-sensitized nickel oxide (NiO) photocathodes. The NiO films were co-sensitized with a molecular dye and a proton reducing catalyst from the family of [FeFe](bdt)(CO)_6_ (bdt = benzene-1,2-dithiolate) complexes. Two dyes were used: an organic push–pull dye denoted E2 with a triarylamine–oligothiophene–dicyanovinyl structure and a coumarin 343 dye. Upon photo-excitation of the dye, a clear spectroscopic signature of the reduced catalyst is observed a few picoseconds after excitation in all co-sensitized NiO films. However, kinetic analysis of the transient absorption signals of the dye and reduced catalyst reveal important mechanistic differences in the first reduction of the catalyst depending on the co-sensitized molecular dye (E2 or C343). While catalyst reduction is preceded by hole injection in NiO in C343-sensitized NiO films, the singly reduced catalyst is formed by direct electron transfer from the excited dye E2* to the catalyst in E2-sensitized NiO films. This change in mechanism also impacts the lifetime of the reduced catalyst, which is only *ca.* 50 ps in E2-sensitized NiO films but is >5 ns in C343-sensitized NiO films. Finally, the implication of this mechanistic study for the development of better co-sensitized photocathodes is discussed.

## Abstract 2

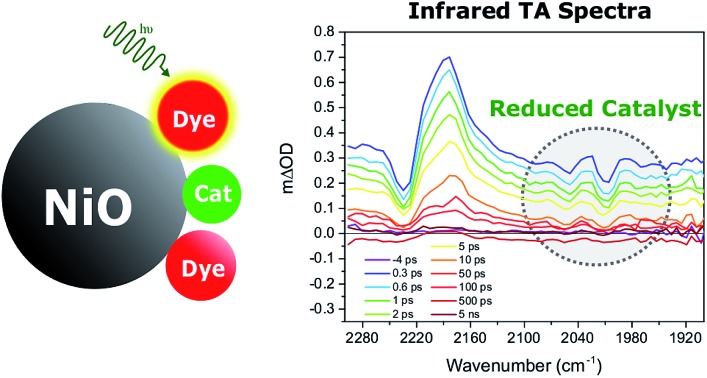
Co-sensitization of molecular dyes and catalysts on semiconductor surfaces is a promising strategy to build photoelectrodes for solar fuel production.

## Introduction

Dye sensitized solar fuel devices (DSSFDs) for conversion of water to hydrogen (H_2_) and oxygen (O_2_) gases suggest a practical solution for storage of solar energy.^1^ Feasibility of such a technology has already been demonstrated with a few reported devices, with photoanodes for water oxidation and photocathodes for hydrogen reduction.^2^–^12^ Most of these photoelectrodes consist of photosensitizers and/or molecular catalysts anchored on transparent semiconductor materials. Typically, n-type titanium dioxide (TiO_2_) is used for the photoanode for water oxidation and p-type nickel oxide (NiO) for the photocathode for proton reduction. While several photoanodes based on TiO_2_ have been reported in the literature,^4^,^6^,^9^,^13^ there are much fewer examples for the photocathode for H_2_ production based on NiO.^2^,^3^,^6^–^12^ The latter often shows poorer performance, hence limits the overall efficiency of the complete DSSFD device. Comparatively, less is also understood on the charge transfer dynamics between the photosensitizer, the molecular catalyst and the NiO semiconductor material.

**Fig. 1:**
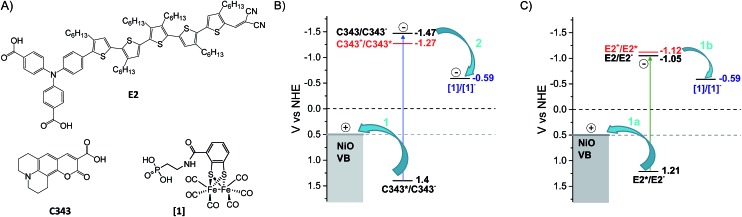
Chemical structures, reduction potentials and investigated charge transfer processes between the NiO semiconductor, the molecular dye and catalyst. (A) Molecular structures of the dyes E2, coumarin 343 (C343) and the catalyst **[1]**. (B) The potentials for reductive quenching (arrow 1) of the dye C343 by hole transfer to the NiO valence band, and subsequent oxidation of the C343^–^ anion (arrow 2) by the catalyst **[1]**, as well as for oxidative quenching of C343 by the catalyst (red digits); the proposed mechanism (arrows) for reduction of the catalyst **[1]** as shown in our previous work using UV-vis transient absorption spectroscopy.^17^ (C) Potential diagram corresponding to B, but with the E2 dye, and investigated charge transfer processes (arrows 1a and 1b) investigated in this study.

Upon illumination of the photocathode, photon absorption by the dye is believed to result in hole injection into NiO to form an interfacial charge-separated state with a reduced dye. From the latter, the molecular catalyst that is important for the photocatalytic process is reduced by another electron transfer step. Efficient photo-induced catalysis imposes several kinetic constraints to the photocathode system: first, electron transfer between the reduced dye and the catalyst must be faster than recombination of the charge-separated state; second, singly reduced catalysts must live long enough to engage in the next step of the catalytic cycle and thus propagate catalysis. To fulfil these two requirements, several strategies have been explored.^12^ One approach is to have the NiO-immobilized dye covalently linked to the molecular catalyst,^10^,^14^ thus offering control over the electronic coupling between the dye and catalyst. In such an ensemble, rapid intramolecular electron transfer between the reduced dye and the catalyst is enabled. However, such a design is synthetically demanding. An alternative approach less challenging in terms of synthesis is to co-adsorb the dye and the catalyst on the semiconductor surface. This approach introduces modularity to the system and thus allows tuning of the dye/catalyst couple and its ratio. In addition, the introduction of an antenna function is enabled. For this design to function, efficient charge hopping between the dye and catalyst on the semiconductor surface is required. Recently, our group demonstrated that co-adsorption of dye and molecular catalyst is indeed a promising approach for the development of photocathodes for hydrogen reduction. In several studies, we showed that molecular catalysts could be reduced upon photoexcitation of dye molecules co-adsorbed on NiO.^15^–^17^ Importantly, we also reported the first DSSFD for H_2_ production based on co-adsorption of a coumarin dye (C343) and a proton reducing FeFe catalyst [**1**] on NiO (Fig. 1).^17^

In the present study, we address the viability of the dye/catalyst co-adsorption approach as a reliable strategy for catalyst activation on surface in the absence of sacrificial electron donor. By varying the nature of the dye co-adsorbed with the proton reducing FeFe catalyst **[1]** on NiO, we investigate how the reduction of **[1]** and its lifetime can be controlled. Here, the organic push–pull dye, E2, with a triphenylamine donor group and dicyanovinyl acceptor group, was chosen as sensitizer and compared with the C343 dye used in our previous work (Fig. 1).^18^ Upon photoexcitation, both dyes give rapid and efficient hole injection into NiO,^18^,^19^ and the resulting reduced dyes are thermodynamically able to reduce the catalyst (Fig. 1).^20^ Thus, using the same preparation conditions, NiO films were sensitized with either the dye E2 or C343, and subsequently the catalyst **[1]** was co-adsorbed.^17^

A major challenge in monitoring electron transfer processes between dye and molecular catalyst on semiconductor surfaces by transient absorption spectroscopy is often the overlapping signals in visible transient absorption experiments. The ground-state absorption spectrum of the E2 dye largely overlaps with the band of the reduced catalyst reported in the visible at around 510 nm.^20^ Hence, it is difficult to detect reduction of **[1]** and quantify the reduced species in the visible transient absorption experiments, which also exhibits lower extinction coefficients than the E2 dye in the visible region.^18^,^20^ Instead, the E2, C343 dyes and the catalyst **[1]** possess distinct reporter groups in the infrared, *i.e.* the dicyanovinyl group (E2) and the carbonyl groups (**[1]** and C343) (see Fig. 1). Recently, Gibson and co-workers showed that vibrations of the cyanovinyl group in the push–pull dye P1 – a dye that is analogous to E2 – are very sensitive to changes in electron density and hence could be used to monitor charge transfer processes in P1-sensitized NiO films.^21^ As for the catalyst **[1]**, its IR spectra and the ones of its reduced states are readily distinguished in the carbonyl region (2100–1800 cm^–1^).^20^ Thus, in the present study, femtosecond mid-infrared transient absorption spectroscopy is used, for the first time, to monitor reduction of catalyst upon dye excitation in co-sensitized NiO films. We give direct evidence of the one-electron photo-reduction of **[1]** in co-sensitized NiO films, **[1]**|E2|NiO and **[1]**|C343|NiO. We also report on the mechanisms leading to catalyst photo-reduction, which we found are very different for the two co-sensitized dyes (E2 or C343). This strongly impacts the yield and lifetime of the singly reduced catalyst, **1^–^**, which ultimately may impede the photocatalytic function of the co-sensitized material in a solar fuel device.

## Results and discussion

### Optical and IR characterization of the sensitized films

**Fig. 2:**
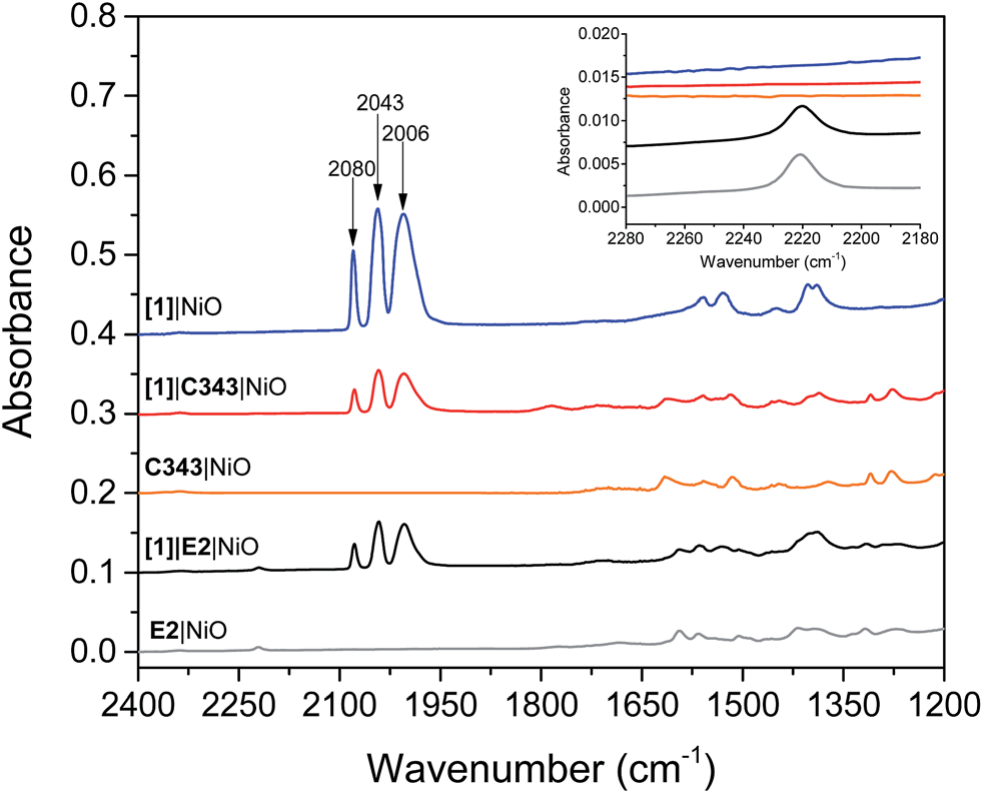
FTIR absorption spectra of the co-sensitized NiO films, **[1]**|E2|NiO (black) and **[1]**|C343|NiO (red). The reference sensitized films, **[1]**|NiO (blue), E2|NiO (gray), C343|NiO films (orange), are also shown. The spectra have been offset by 0.1 absorbance units. Inset in panel B shows the region of the *ν*_C≡N_ stretch of the E2 dye.

**Fig. 3:**
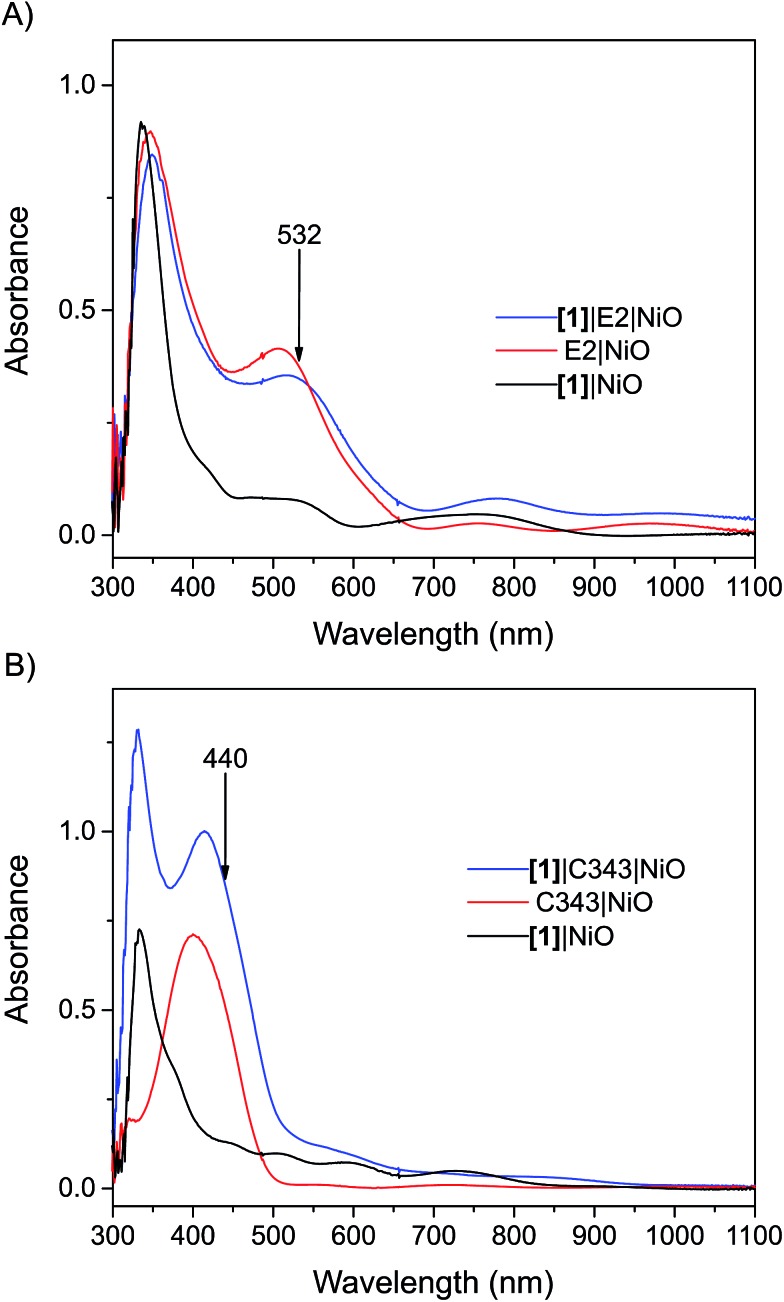
UV-vis absorption spectra of the sensitized NiO films: (A) the **[1]**|E2|NiO co-sensitized NiO film (blue), E2|NiO reference film (red), **[1]**|NiO reference film (black); (B) the **[1]**|C343|NiO co-sensitized NiO film (blue), C343|NiO reference film (red), **[1]**|NiO reference film (black). The arrows indicate the excitation wavelength used in the femtosecond transient absorption experiments. All the spectra have been corrected for the absorption of NiO. The UV-vis absorption spectra before subtraction of the NiO absorption are shown in the Fig. S1 in the ESI.†

Mesoporous NiO films on CaF2 substrates were sensitized by the dyes and catalysts, as described in the Experimental section. The Fourier transform infrared (FTIR) spectra of the co-sensitized NiO films, denoted **[1]**|E2|NiO and **[1]**|C343|NiO, and the reference films designated by **[1]**|NiO, E2|NiO and C343|NiO are shown in Fig. 2. Clear peaks at 2006, 2043 and 2080 cm^–1^ corresponding to the vibrational stretches of the carbonyl groups of **[1]** are observed in the spectra of the co-sensitized films, **[1]**|E2|NiO and **[1]**|C343|NiO, and the reference **[1]**|NiO, which confirms the attachment of **[1]** on the NiO surface.^16^,^17^,^20^ Evidence for the attachment of the E2 dye on the NiO surface for the **[1]**|E2|NiO and E2|NiO films is provided by a peak at 2221 cm^–1^ assigned to the C≡N stretch vibrations of the E2 dye (see inset of Fig. 2).^21^,^22^ For the C343-sensitized films, vibrational peaks at *ca.* 1612 cm^–1^ and 1310 cm^–1^ ascribed to the C343 dye attest the anchoring of C343 on the NiO films (Fig. S2 in the ESI†). The ultraviolet-visible (UV-vis) absorption spectra of the co-sensitized NiO films further confirm the presence of the dyes (E2 or C343) and catalyst **[1]** on the NiO surface (Fig. 3). The UV-vis absorption spectrum of **[1]**|C343|NiO clearly shows mixed features from C343 and **[1]** with a broad dye absorption centred at ∼410 nm and a weak shoulder around 500 nm due to catalyst absorption (Fig. 3B).^17^,^20^ In contrast, for the E2-sensitized films, the E2 absorption bands at ∼500 nm and at ∼350 nm dominate the absorption spectra and overlap largely with the weak absorption bands of **[1]** at 500 nm and <350 nm (Fig. 3A).^18^,^20^ This makes it difficult to detect the presence of the catalyst in **[1]**|E2|NiO from the UV-vis spectrum, although it is clearly seen in the infrared one (Fig. 2).

One important parameter to ensure a relevant comparison between the co-sensitized **[1]**|E2|NiO and **[1]**|C343|NiO films is a similar dye to catalyst ratio. Similar dye to catalyst ratios of 1 : 1 were found in both co-sensitized sensitized **[1]**|E2|NiO and **[1]**|C343|NiO films. The concentration of dye and of **[1]** were also similar between the two types of samples (*cf.* the similar intensity of the carbonyl bands in Fig. 2). For the C343-sensitized films, the dye to catalyst ratio was estimated by comparing the intensities of the vibrational peaks at 2043 cm^–1^ for **[1]** and at 1309 cm^–1^ for C343 for **[1]**|C343|NiO with the ones obtained for a 1 : 1 **[1]**|C343 solution.^16^ In a similar way, for the E2-sensitized films, comparison of the intensity of the vibrational peak at 2043 cm^–1^ for **[1]** in **[1]**|E2|NiO with a solution of **[1]** allowed us to estimate the concentration of **[1]** in **[1]**|E2|NiO. The concentration of attached E2 in **[1]**|E2|NiO was evaluated from the intensity of the E2 absorption band at 500 nm in the UV-vis after subtraction of the NiO absorption.^18^ In both comparisons, we assumed that the extinction coefficients of the selected bands for E2, C343 and **[1]** were similar in solution and when attached on NiO.

### Photoinduced formation of the singly reduced catalyst

**Fig. 4:**
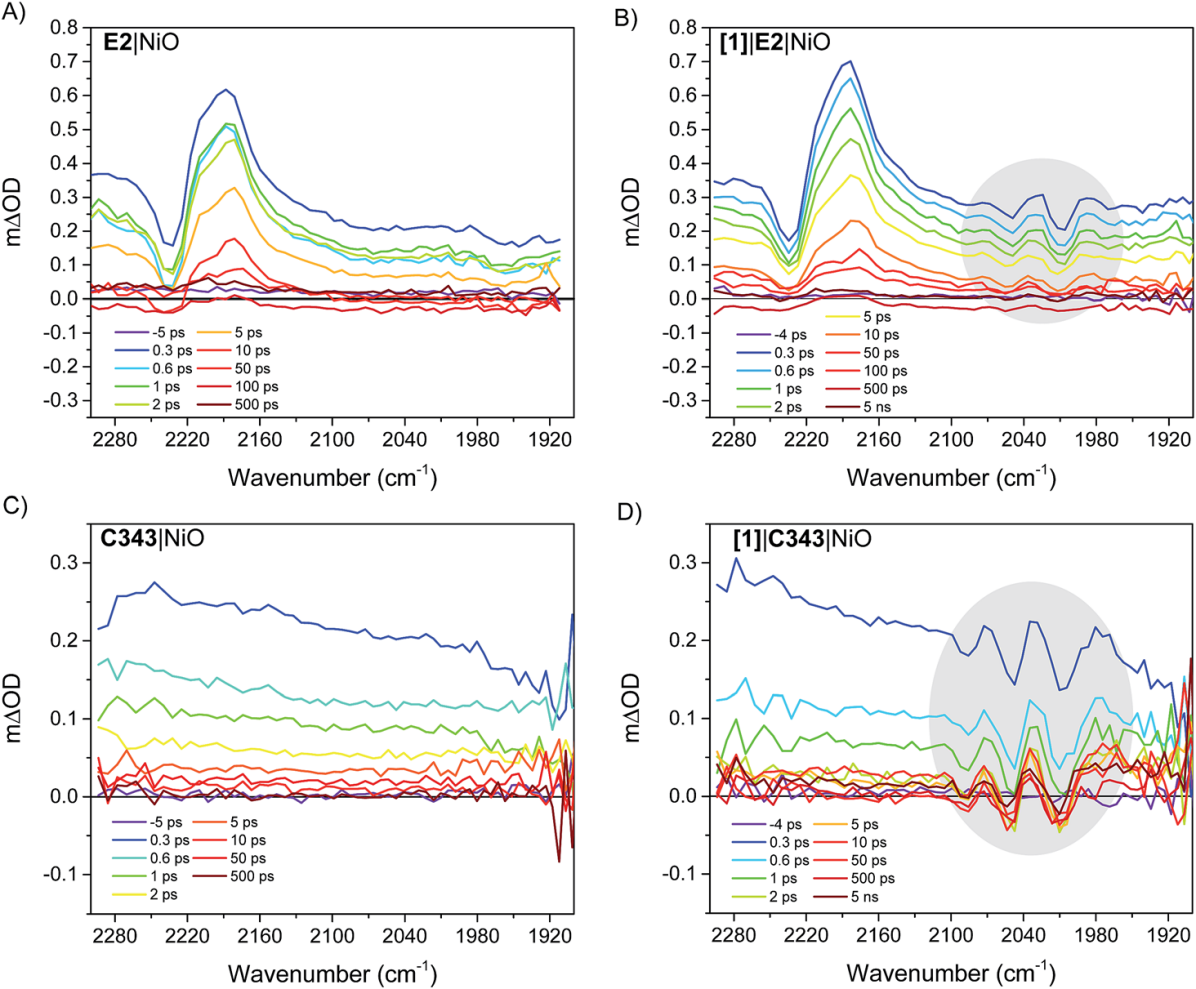
(Right panel): infrared TA spectra showing the reduction of the catalyst **[1]***via* the carbonyl bands (gray areas) in the co-sensitized **[1]**|E2|NiO and **[1]**|C343|NiO films. (Left panel): infrared TA spectra of the sensitized E2|NiO and C343|NiO films without catalyst **[1]**. The solvent was propylene carbonate. The E2|NiO and **[1]**|E2|NiO films were excited at *λ*_exc_ 532 nm with 200 nJ pulse intensity. The C343|NiO and **[1]**|C343|NiO films were excited at 440 nm with 530 nJ pulse intensity.

Fig. 4 right panel shows the femtosecond mid-infrared transient absorption (TA) spectra obtained for the co-sensitized **[1]**|E2|NiO and **[1]**|C343|NiO films in propylene carbonate (PC), after excitation at 532 nm and 440 nm, respectively. At both excitation wavelengths (440 nm and 532 nm), the absorption of **[1]** is low compared to that of the dyes, E2 and C343 (*ε*(E2) = 38 000 M^–1^ cm^–1^*vs. ε*(**[1]**) = 260 M^–1^ cm^–1^ at 532 nm, *ε*(C343) = 15 100 M^–1^ cm^–1^*vs. ε*(**[1]**) = 1280 M^–1^ cm^–1^ at 440 nm) and hence mainly the dye (*i.e.* E2 or C343) is excited.^18^,^20^,^23^ The TA spectra of the dye-sensitized films without catalyst, E2|NiO and C343|NiO, are also shown as references in the left panel of Fig. 4. For **[1]**|E2|NiO and E2|NiO films, a ground-state bleach at 2232 cm^–1^ of the C≡N stretch of E2 and two positive absorption bands at 2210 cm^–1^ and 2181 cm^–1^ are immediately seen after excitation of E2. These features are consistent with hole injection from excited E2* into NiO and the formation of the charge separated state E2^–^|NiO^+^. Gibson and co-workers reported similar spectral features in the transient IR absorption spectra of P1-sensitized NiO films.^21^ Similarly to the E2 dye, P1 consists of a triphenylamine donor and two dicyanovinyl acceptor groups. Following irradiation, the electron density in E2 shifts away from the triphenylamine group and localizes on the cyano groups. After hole injection, this excess electron density remains on the cyano groups, giving rise to the observed transient bands at lower energy. More interestingly, in addition to the spectral features assigned to the E2 dye, **[1]**|E2|NiO shows clear bleaches of the CO bands of **[1]** at 2006 cm^–1^, 2049 cm^–1^ and positive peaks at 2074 cm^–1^, 2030 cm^–1^ and 1988 cm^–1^ (gray area in Fig. 4B). These spectral features closely resemble the ones observed for the singly reduced catalyst [Fe_2_(bdt)(CO)_6_]^–^.^20^

**Fig. 5:**
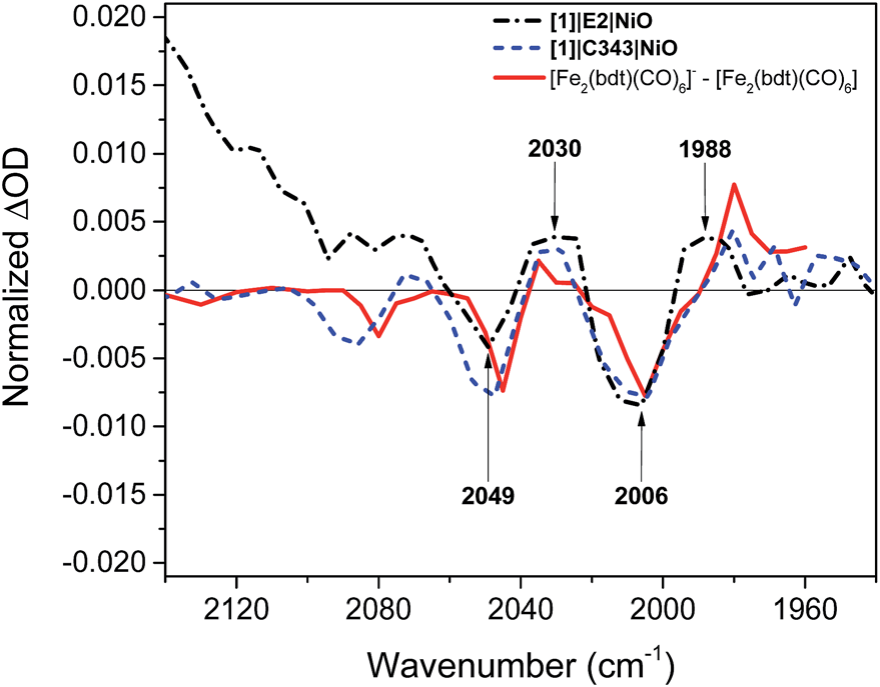
Comparison of the IR transient absorption spectrum of the **[1]**|E2|NiO film 1 ps after excitation at 532 nm (black), the **[1]**|C343|NiO film 1 ps after excitation at 440 nm (blue), and the IR transient absorption spectrum of [Fe_2_(bdt)(CO)_6_]^–^–[Fe_2_(bdt)(CO)_6_] obtained by reduction of [Fe_2_(bdt)(CO)_6_] with flash-quench generated [Ru(dmb)_3_]^+^ (ref. 18) (red).

Fig. 5 compares the IR TA spectrum of **[1]**|E2|NiO at 1 ps and the IR spectrum of the difference [Fe_2_(bdt)(CO)_6_]–[Fe_2_(bdt)(CO)_6_]^–^.^20^ The spectra are nearly identical and confirm the formation of the reduced catalyst **1^–^** in co-sensitized **[1]**|E2|NiO films. When changing the E2 dye for the dye C343, a clear spectroscopic signature of the reduced **1^–^** is also observed in **[1]**|C343|NiO, in agreement with previous UV-vis transient absorption experiments.^17^ The ground state bleaches at 2086 cm^–1^, 2047 cm^–1^ and 2006 cm^–1^ and positive peaks at 2073 cm^–1^, 2034 cm^–1^ and 1980 cm^–1^ due to **1^–^** are more pronounced than in the sample with E2, due to the absence of overlapping bands from the dye. Note that for **[1]**|C343|NiO and C343|NiO, we do not expect any transient signal assigned to the dye excited state C343* or reduced state C343^–^ due to the absence of characteristic vibration bands of C343 in the probe wavenumber range (Fig. 2). Instead, formation of C343^–^ by ultrafast (≈200 fs) hole injection into NiO has been verified by UV-vis TA experiments.^17^ A broad background IR absorption is observed in the TA spectra of all the dye-sensitized NiO films that decays within ∼13–16 ps for the E2-sensitized NiO films and ∼2.5–5 ps for C343-sensitized NiO (Fig. S3 and S4†). This background absorption may be assigned to relaxation of holes injected into NiO and/or to thermal effects. This overall broad background is absent in the TA spectra of **[1]**|NiO films in which no hole injection occurs (Fig. S5 and S6†). This suggests that the broad background absorption in the dye-sensitized NiO films is due to injected holes into NiO that thermalize or otherwise relax to hole states with smaller mid-IR signal. Finally, upon excitation at 532 nm and at 440 nm, the TA spectra of **[1]**|NiO show negligible signals from reduced **1^–^**. Formation of reduced **1^–^** by direct excitation of **[1]** in **[1]**|E2|NiO and **[1]**|C343|NiO can be excluded. In other words, the co-sensitized dye is essential to the reduction of **[1]** in both **[1]**|E2|NiO and **[1]**|C343|NiO films.

### Formation dynamics of the singly reduced catalyst

**Fig. 6:**
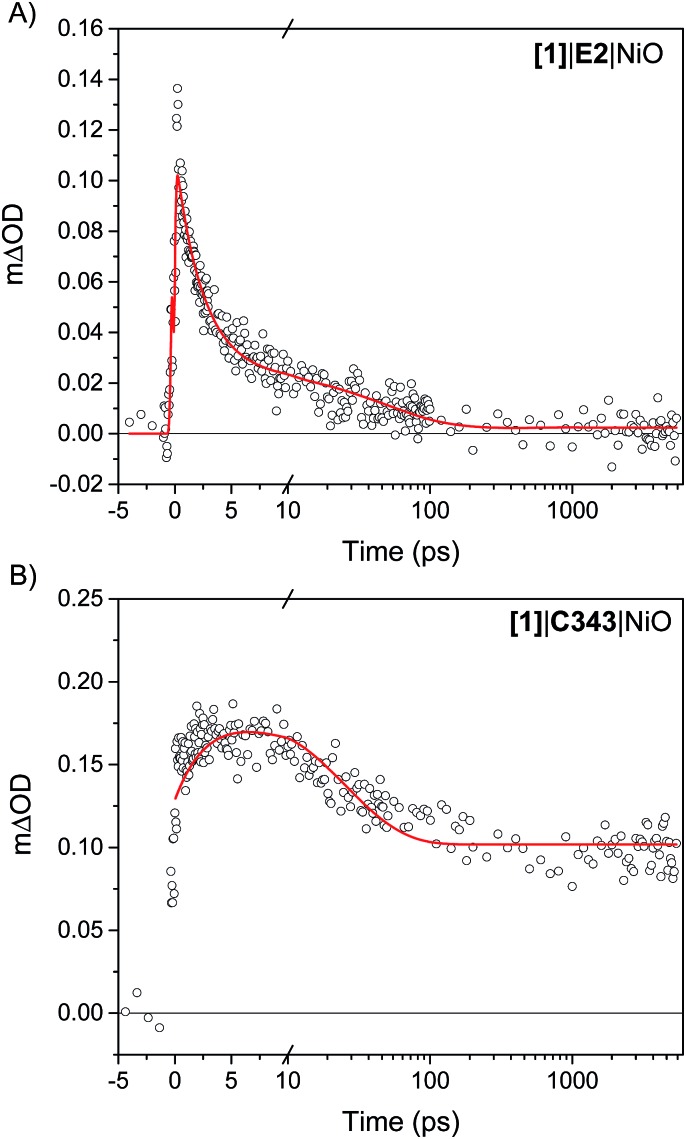
Kinetics of reduction of **[1]** upon photoexcitation of the co-sensitized dye in (A) **[1]**|E2|NiO and (B) **[1]**|C343|NiO films. For **[1]**|E2|NiO, the kinetics of reduction of **[1]** is obtained from the difference between the transient absorption signals of **1^–^** and **[1]** taken at 2024 cm^–1^ and 2006 cm^–1^,respectively. In a similar way, the kinetics of reduction of **[1]** in **[1]**|C343|NiO is generated from the difference of the sum of transient absorption signals of **1^–^** taken at 2072 cm^–1^ and 2034 cm^–1^ and the sum of the transient signals of **[1]** taken at 2053 cm^–1^ and 2010 cm^–1^. Two peaks difference, instead of one peak difference was used for **[1]**|C343|NiO to improve the signal to noise ratio. The red lines are fits with three exponentials to the experimental data (Table 1).

The kinetics of the transient absorption signals in the carbonyl bands of **[1]** (gray areas in Fig. 4) provides further mechanistic insights into the formation of reduced catalyst. Fig. 6 compares the kinetics of reduction of **[1]** in **[1]**|E2|NiO and **[1]**|C343|NiO films. Significant kinetic differences are observed between **[1]**|E2|NiO and **[1]**|C343|NiO samples. With C343, a rise in the kinetics is observed indicating a built-up of reduced catalyst **1^–^** within *ca.* 3 ps, while reduction of **[1]** is instrument-limited in **[1]**|E2|NiO (*i.e.* no rise). Further, in **[1]**|C343|NiO, the signal of reduced **1^–^**, despite a slight decay due to partial recombination with holes in NiO, persists for the entire duration of the experiment, up to 5 ns (Fig. S7†). In contrast, the signal of reduced **1^–^** rapidly disappears within 100 ps for **[1]**|E2|NiO (Fig. 6A). Such differences suggest distinctly different reduction paths of **[1]** in **[1]**|E2|NiO and **[1]**|C343|NiO (Table 1).

**Table 1:** Lifetimes from a three-exponential fit to the kinetics of reduction of **[1]** in Fig. 6 for **[1]**|E2|NiO and **[1]**|C343|NiO

	*τ*_1_ (A1)	*τ*_2_ (A2)	*τ*_3_ (A3)
**[1]**|E2|NiO	2.4 ps (77%)	50 ps (21%)	>5 ns (2%)
**[1]**|C343|NiO	3.4 ps (–27%)	24 ps (37%)	>5 ns (36%)

Looking now at the hole injection dynamics of the dye, both dyes show hole injection dynamics unchanged by the presence of **[1]**. For **[1]**|E2|NiO, the signal at 2180 cm^–1^ attributed to the charge separated E2^–^|NiO^+^ decays on a similar time scale for **[1]**|E2|NiO and E2|NiO within 100 ps (Fig. S8 and Table S1†).^21^ These traces have kinetics very similar to the ones obtained by Sheibani *et al.*^18^ in visible transient absorption experiments for E2|NiO films. For the C343-sensitized films, additional TA spectra were collected in the region 1700–1500 cm^–1^ where C343 possesses C=O vibrations (Fig. S9 and S10†). Co-adsorption of **[1]** does apparently not perturb hole injection of excited C343* into NiO. The ground state bleach of C343 at 1616 cm^–1^ recovers with nearly identical time constants in C343|NiO and **[1]**|C343|NiO films, *i.e.* 0.3 ps and 8.5 ps (Fig. S10 and Table S2†). These time constants are within experimental error the same as the ones obtained in visible transient absorption experiments for C343-sensitized NiO films by Morandeira *et al.*^19^ The 0.3 ps is assigned to hole injection from the excited C343* to the valence band of NiO while the 8.5 ps component corresponds mainly to partial recombination of the charge-separated state with holes in NiO.

As mentioned earlier, charge transfer reactions in co-sensitized **[1]**|C343|NiO films have been previously studied by our group using femtosecond and nanosecond transient absorption in the visible.^17^ Hole injection dynamics in **[1]**|C343|NiO was found unperturbed by the presence of **[1]**. A half-lifetime of *ca.* 6 ps for the formation of reduced **[1]** and a lifetime of reduced **[1]** of 2 μs to 20 ms were reported. Here, our results are consistent with the ones obtained by Antila *et al.*^17^ (*vide supra*). They confirm the proposed mechanism for reduction of **[1]** in co-sensitized **[1]**|C343|NiO (Fig. 1), *i.e.* hole injection from the excited C343 to NiO followed by surface electron transfer from the reduced C343^–^ to the catalyst **[1]**. For **[1]**|E2|NiO films, the immediate signal of reduced **1^–^** raises the question whether the NiO plays an active role in the reduction of **[1]**. Two scenarios may be envisaged: in the first one, reduced **1^–^** is formed by direct electron transfer (ET) from the excited dye E2*; the second scenario is a two-step mechanism with hole injection by E2* into NiO and subsequent ultra-fast surface electron transfer from the reduced dye E2^–^ to the catalyst **[1]**. As discussed below, our data suggest the first scenario. Moreover, control experiments on co-sensitized ZrO_2_ films with E2 and **[1]** in a reasonably comparable dye to catalyst ratio of *ca.* 1 : 2 give very similar results (Fig. S12–S15, Table S3†). For energetic reasons, no hole or electron injection can occur into ZrO_2_. Therefore, reduction of **[1]** must occur directly by the excited E2* also in this sample. Looking at the energetics in Fig. 1, the excited E2* has indeed enough reducing power to reduce **[1]**, *i.e.* Δ*G*_0_ = –0.53 eV.^18^

### Discussion

**Scheme 1:**
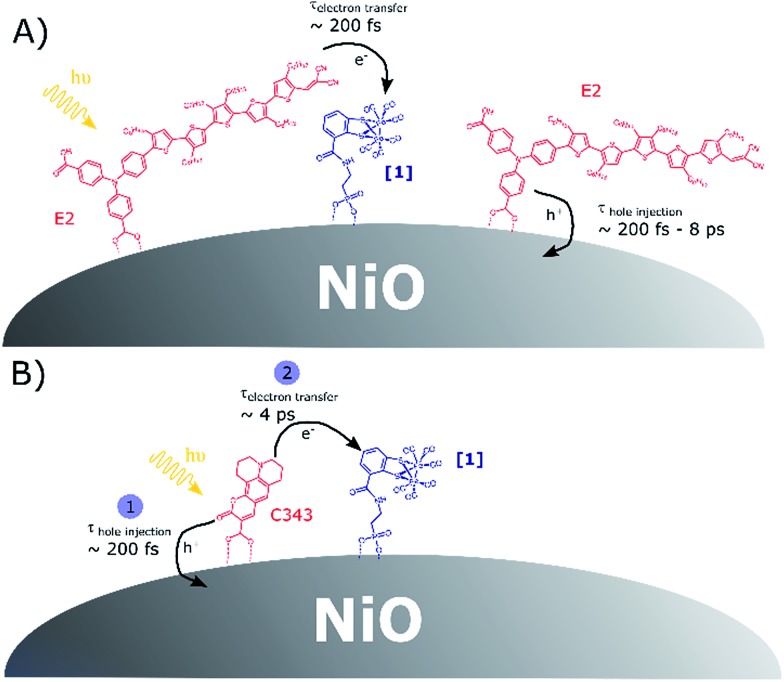
(A) Competitive photoinduced charge transfer processes in co-sensitized **[1]**|E2|NiO films; (B) sequence of photoinduced electron transfer processes leading to catalyst reduction in co-sensitized **[1]**|C343|NiO films, in agreement with the data in ref. 17.

Scheme 1 presents the proposed mechanisms for reduction of **[1]** in NiO films co-sensitized with the catalyst **[1]** and a dye (E2 or C343). For **[1]**|C343|NiO films, reduction of **[1]** takes place *via* a sequence of charge transfer processes, with hole injection from the excited C343* to NiO followed by surface electron transfer from the reduced C343^–^ to **[1]**. In contrast, in NiO films co-sensitized with E2 and catalyst **[1]**, we propose that two parallel photo-induced electron transfer processes co-exist. Hole injection from the excited E2* into the valence band of NiO competes with the reduction of **[1]** by direct electron transfer from E2* to **[1]**. The latter path is presumably followed by only a small fraction of the excited E2*. These findings may seem surprising, but are supported by the following observations.

The first is the absence of rise in the kinetics of catalyst reduction in **[1]**|E2|NiO, although hole injection is resolvable. Additional femtosecond transient UV-vis absorption spectra of E2|NiO and **[1]**|E2|NiO films confirm that hole injection in NiO by E2* does indeed take place in both E2|NiO and **[1]**|E2|NiO (Fig. S16 and S17†).^18^

The second observation is the low quantum yield of catalyst reduction. The amplitude of the TA bleach at *ca.* 2049 cm^–1^ for **[1]** is comparable to the E2 TA bleach at *ca.* 2230 cm^–1^ (Fig. 4), whereas in the ground state FTIR, the carbonyl bands are instead about 25 times stronger than the E2 dicyanovinyl bands (Fig. 3). From this comparison, and assuming that the TA bleach intensity is proportional to the ground state absorption, it seems that only about 4% of the excitations lead to reduction of **[1]**, while the remaining *ca.* 96% result in E2-NiO charge separation. Further, as mentioned earlier, the kinetics of the E2 dye TA signal is not significantly affected by the presence of **[1]** (Fig. S8 and Table S1†).

The third observation is the much faster decay of **1^–^** in **[1]**|E2|NiO compared to **[1]**|C343|NiO (Fig. 6). This points to a different recombination mechanism in the two samples, *i.e.* with **1^–^** recombines with E2^+^ in the first case and with NiO holes in the case of **[1]**|C343|NiO.

From the small fraction of excited E2* that engage into direct electron transfer to **[1]**, a charge-separated state **1^–^**|E2^+^ forms on the NiO surface. One question is whether we should expect an infrared signature for the oxidized dye E2^+^ that differs from the reduced dye E2^–^ formed by hole injection. To test this, we attached E2 to a TiO_2_ film and measured its fs TA spectra in the infrared. The E2 dye effectively injects electrons in the conduction band of TiO_2_, as indicated by the broad absorption band in the entire probe region (Fig. S18†).^24^,^25^ In the cyano region, the spectral features remain nearly identical to the ones observed for the charge-transfer excited state in solution (Fig. S19†) and the E2-sensitized ZrO_2_ film (Fig. S12†), with a down-shifted IR band. From this control experiment, we can conclude that the oxidized dye E2^+^, the charge-transfer excited state of the E2 dye, and the reduced dye E2^–^ give rise to similar, but not identical, transient signals in the cyano region in the infrared. This can be rationalized by similar bond orders in the excited state E2*, the reduced E2^–^ and the oxidized E2^+^. Previous work by Sun and co-workers showed indeed, that the excited state and the reduced state of the push–pull dye P1 – a dye that is analogous to E2 – possess an excess electron density in antibonding molecular orbitals while in the oxidized dye, bonding molecular orbitals that are delocalised become electron deficient.^22^ This may result in a similar bond order and stiffness of the CN vibration.

Our study reports the possibility to directly monitor ultra-fast, intramolecular electron transfer on semiconductor surfaces by mid-IR TA spectroscopy. Besides the novelty in the use of ultra-fast mid-IR spectroscopy for such characterization, an interesting outcome of this study is the surprising change in mechanism for catalyst reduction upon modification of the co-sensitized dye. While for **[1]**|C343|NiO, our results confirm the multi-step mechanism proposed by Antila *et al.*, we show herein that switching the dye C343 to E2 impacts the reaction path for the formation of the reduced catalyst and its lifetime. In **[1]**|E2|NiO films, most of the dyes E2 are insensitive to the presence of **[1]** and engage in hole injection into the valence band of NiO, followed by charge recombination before any significant further transfer of electrons to **[1]**. Only few excited E2* reduce **[1]** by direct electron transfer. The resulting oxidized E2^+^ is unable to inject holes into NiO and instead recombines quickly with the reduced **1^–^**, hence its short lifetime. As shown in Fig. 1, both C343 and E2 possess suitable redox potentials to reduce **[1]**, either from the excited state or from the reduced state. However, one may note that in contrast to C343, the excited state for E2 is more reducing than its reducing state. Nevertheless, the driving force for reduction of **[1]** by the anion E2^–^ is quite large (Δ*G*_0_(E2^–^ → **[1]**) = –0.46 eV). Thus, it seems unlikely that the main reason for such a mechanistic change lies only in the energetics. Instead, we propose that such differences between **[1]**|C343|NiO and **[1]**|E2|NiO arise from different arrangements of the dye and catalyst on the NiO surface. For the C343 dye, X-ray reflectometry experiments have shown that C343 dyes adsorbed onto amorphous TiO_2_ orient themselves with a relative angle of 61.1° to the TiO_2_ surface.^26^ Although the NiO surface may be different in term of charge density, one may assume that the C343 dyes adopt a similar orientation on NiO surfaces and are tilted relative to the NiO surface. Such an orientation of the C343 dyes onto the NiO surface may enable π-stacking between the co-sensitized C343 and catalyst **[1]** and hence favour electronic interactions. This could explain the rapid catalyst reduction (*τ* ≈ 4 ps) in **[1]**|C343|NiO that competes favourably with recombination of the reduced C343^–^ with holes. In contrast, for the E2 dye, previous work by Sheibani *et al.* suggested that the dye E2 lies down on the NiO surface, with the dicyanovinyl acceptor groups very close to the NiO surface.^18^ Such an arrangement of the E2 dyes onto the NiO surface would prevent π-stacking to occur between E2 and **[1]** in **[1]**|E2|NiO samples. Most of the E2 dyes may then be unable to reduce **[1]**, because they are not close enough to react before E2^–^ recombines with holes. Long-range surface electron hopping is presumably required for catalyst reduction in **[1]**|E2|NiO, but this may be too slow and inefficient due to poor electronic interactions. On the other hand, few E2 dyes (*i.e.* ∼4%) get close enough to **[1]** that they can undergo direct electron transfer to **[1]**. Hypothetically self-assembly of the E2 dyes and **[1]** could be visible in the ground-state absorption spectra if the majority of the dyes did self-assemble with the catalyst. However, as mentioned earlier, here, this interaction would only concern a small fraction of the E2 dyes anchored (*i.e.* ∼4%). In addition, E2 absorbs strongly in the UV-vis region where **[1]** has only weak and overlapping absorption bands. This makes it nearly impossible to distinguish any interaction between E2 and **[1]** from the ground-state absorption spectra. To shed light on the influence of the dye-catalyst arrangement in co-sensitized photocathodes, we are currently investigating the possibility of embedding dye-sensitized NiO films in ALD Al_2_O_3_ layers prior to attachment of the catalyst.

In summary, our work suggests that sufficient electronic interaction between the dye and catalyst *via e.g.* π-stacking is required for efficient electron transfer between dye and catalyst in co-sensitized photoelectrodes. This presents a challenge for the co-sensitization strategy. Further work must address these issues, if our results with E2 are general for push–pull organic dyes with catalysts, and explore strategies on how to control the self-assembly of dye and catalyst onto surfaces. This may be done by implementing in the dye design for example, substituents enabling π-stacking between dye and catalyst.

## Conclusions

We have employed femtosecond mid-infrared transient absorption spectroscopy to follow charge transfer reactions in NiO photocathodes co-sensitized with molecular dyes and catalyst **[1]**. Two types of dyes have been used to initiate the reduction of the catalyst, namely the push–pull dye E2 and coumarin 343. With both dyes, a clear spectroscopic signature of the reduced catalyst was observed upon photo-excitation of the co-sensitized dye. Further kinetic analysis reveal different reaction paths for catalyst reduction in **[1]**|E2|NiO and **[1]**|C343|NiO films. With C343, in agreement with our previous study using optical transient absorption,^10^ a multi-step path leads to the singly reduced catalyst. These are an initial hole injection from the excited C343* into the NiO valence band and second, surface electron transfer from the reduced C343^–^ to the catalyst. In contrast, subpicosecond direct electron transfer from the excited E2* to the catalyst **[1]** produces the singly reduced catalyst **1^–^** in **[1]**|E2|NiO. Concomitant with the change in mechanism comes a drastic decrease of the reduced catalyst's lifetime from >5 ns in **[1]**|C343|NiO to 50 ps in **[1]**|E2|NiO, which precludes any light-induced, second reduction step to occur. We propose that such a mechanistic difference mainly arises from different arrangements of the dye and catalyst on the NiO surface. This mechanistic study highlights the importance of the dye/catalyst packing on the performance of co-sensitized NiO-based photocathodes.

## Experimental details

### Materials

All solvents were used as received without further purification. Propylene carbonate (reagent grade) was purchased from Sigma Aldrich. Both methanol and dichloromethane (spectroscopic grade) were obtained from Merck. The dye coumarin 343 (C343) was purchased from Sigma Aldrich (dye content 97%). The E2 dye with a triarylamine–oligothiophene–dicyanovinyl structure was synthesized according to the procedure reported previously.^18^ Synthesis of the catalyst **[1]** was realized according to our previously reported procedure.^17^,^20^

### Preparation of co-sensitized NiO films

Double-layered nickel oxide (NiO) films were prepared on CaF_2_ windows according to the procedure reported in the literature.^27^ For each set of experiments (*i.e.* sensitization with the C343 or E2 dye), three double-layered NiO films were used. Two of the films were immersed in a saturated-dye bath, *i.e.* a saturated C343 solution overnight for C343-sensitized NiO films or a dichloromethane (DCM) solution of E2 for few hours for E2-sensitized NiO films. The films were then rinsed and their UV-vis (Agilent 8453 UV-vis spectrophotometer) and IR (Bruker 66v/S FTIR spectrometer) absorption spectra were recorded (Fig. 2, 3 and S11†).

Subsequently, one of the dye-sensitized NiO film and one non-sensitized NiO film were immersed in a solution of **[1]** (1.7 mg of **[1]** in methanol) for 48 hours. After 48 hours, both films were rinsed with methanol and their UV-vis and IR absorption spectra were recorded (Fig. S1†). After correction for the NiO background absorption, the optical density of the **[1]**|C343|NiO and C343|NiO and **[1]**|NiO films were ∼0.76, 0.51 and 0.2 respectively at 440 nm, *i.e.* the pump wavelength used in the fs transient absorption measurements. For the experiments using the E2 dye, the absorbance at 532 excitation wavelength of the **[1]**|E2|NiO and E2|NiO films was ∼0.35, and that of the reference sample **[1]**|NiO was ∼0.08. Co-sensitized zirconium oxide (ZrO_2_) films were also prepared as references (no hole injection into the valence band of ZrO_2_). The ZrO_2_ films were prepared on CaF_2_ windows by doctor-blading followed by annealing at 500 °C for 30 min. The obtained ZrO_2_ films were then sensitized following the procedure described above.

### Femtosecond mid-infrared transient absorption spectroscopy

Femtosecond mid-infrared (mid-IR) transient absorption of the sensitized NiO films was performed using a femtosecond transient absorption spectrometer (Helios IR, Ultrafast Systems LLC). The 800 nm output of a Ti:sapphire based amplifier with integrated oscillator and pump lasers (800 nm, 40 fs, 3 kHz, Libra LHE, Coherent Inc.) was split into two beams which were used to pump two TOPAS Primes coupled with frequency mixers (Light Conversion Ltd). This produced a depolarized visible pump pulse (440 nm or 532 nm) and a broad mid-IR probe spectrum. Pump pulse energies were adjusted using a neutral density filter placed prior the sample and varied between 200 nJ at 532 nm (spot size *ca.* 1900 μm^2^) and 530 nJ at 440 nm (spot size *ca.* 3000 μm^2^) depending on the measured sample.

All spectra were recorded in a sealed liquid cell (Specac) with CaF_2_ windows (one of them used as substrate for the sensitized NiO films) and a PTFE spacer of 56 μm path length. In all experiments, propylene carbonate was used as solvent. Propylene carbonate (PC) was chosen as solvent since it does not exhibit any vibrations in the 2300–1900 cm^–1^ infrared probed region and solubility of the dye and catalyst in PC was found negligible.

## Conflicts of interest

We have no conflicts of interest to declare.

## Supplementary Material

Supplementary information
