# Supplementary material for: Direct evidence of catalyst reduction on dye and catalyst co-sensitized NiO photocathodes by mid-infrared transient absorption spectroscopy
Source: Chem Sci. 2018 May 8;9(22):4983–91. doi: 10.1039/c8sc00990b (PMC5989651; doi:10.1039/c8sc00990b)
Supplement: Supplementary file 1 [file SC-009-C8SC00990B-s001.pdf]

Electronic Supplementary Material (ESI) for Chemical Science.
This journal is © The Royal Society of Chemistry 2018

Electronic Supplementary Information

Direct evidence of catalyst reduction on dye and catalyst co-sensitized NiO photocathodes by mid-infrared transient absorption spectroscopy

M. Gilbert Gatty,[a] S. Pullen,[a] E. Sheibani,[b†] H. Tian,[a] S. Ott[a] and L. Hammarström[a]

[a] Physical Chemistry, Department of Chemistry, Ångström Laboratory, Uppsala University, Box 523, 75120 Uppsala, Sweden

[b] Organic Chemistry, Department of Chemistry, Chemical Science of Engineering, KTH, Royal Institute of Technology, Teknikringen 30, 100 44, Stockholm, Sweden

[†] Present address: Department of Chemistry, University of Ishafan, Ishafan 81746-73441, Iran

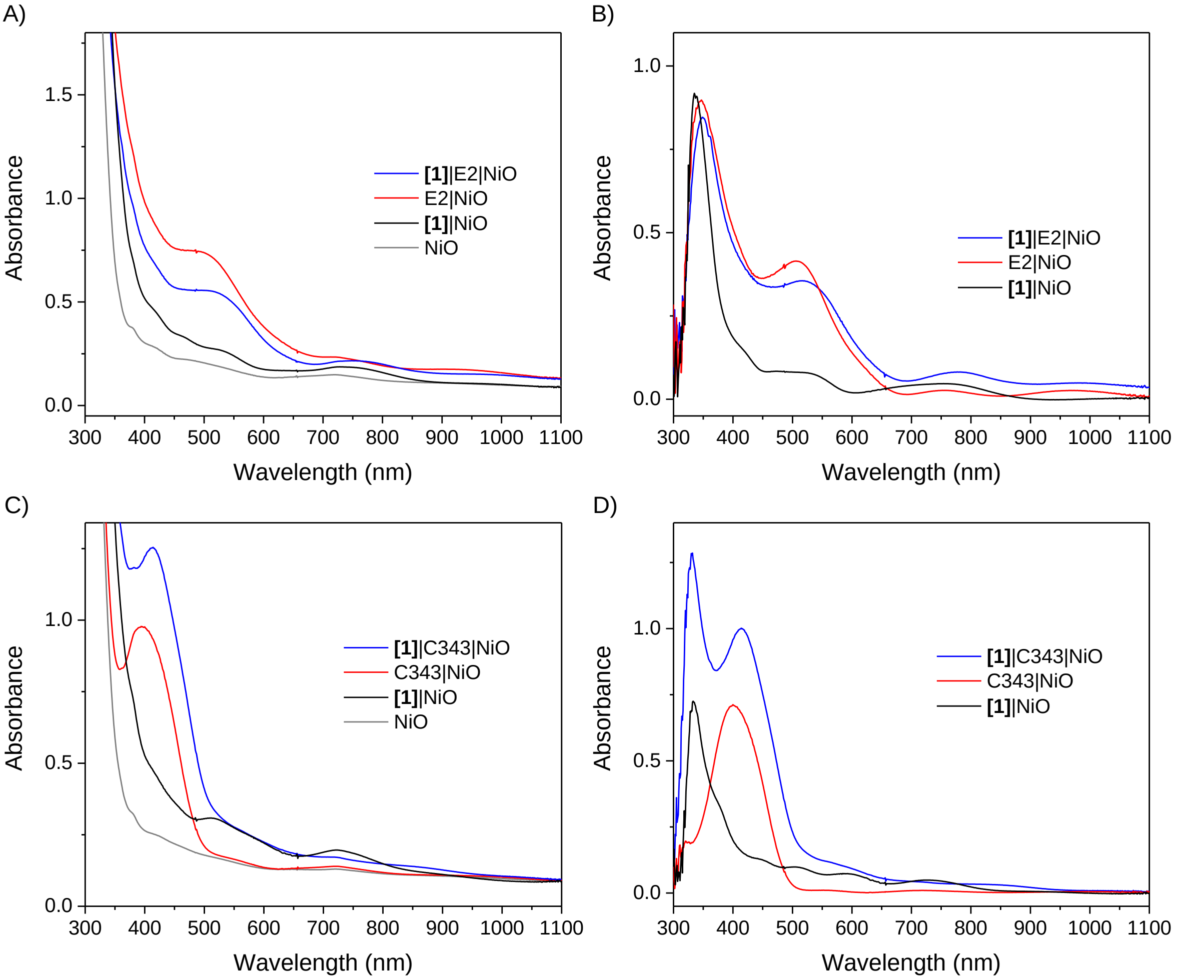

Fig. S1 UV-Vis ground-state absorption spectra of the sensitized NiO films: A) the **[1]**|E2|NiO co-sensitized NiO film (blue), E2|NiO reference film (red), **[1]**|NiO reference film (black), and a NiO film before sensitization, B) the **[1]**|E2|NiO co-sensitized NiO film (blue), E2|NiO reference film (red), **[1]**|NiO reference film (black) after subtraction of the NiO background absorption, C) the **[1]**|C343|NiO co-sensitized NiO film (blue), C343|NiO reference film (red), **[1]**|NiO reference film (black), and a NiO film before sensitization and D) the **[1]**|C343|NiO co-sensitized NiO film (blue), C343|NiO reference film (red), **[1]**|NiO reference film (black) after subtraction of the NiO background absorption.

The E2-sensitized NiO film (E2|NiO) exhibits a broad absorption band at ~500 nm corresponding to a π –π* transition as reported earlier.[1] In contrast, **[1]** on NiO film (**[1]**|NiO) show relatively weak absorption in the visible and no clear absorption bands can be distinguished. From an earlier study, **[1]**|NiO showed a very weak absorption band centered at 450 nm.[2] The broad background absorption from the double-layered NiO film masks the weak absorption bands of **[1]**. When subtracting the background absorption of the double-layered NiO film to the UV-vis absorption spectrum of **[1]**|NiO, a strong absorption band at 340 nm and a weak absorption band centered at ca.

450 nm is observed, similar to those reported for an analogous catalyst [Fe_2(bdt)$(CO)_6$] in solution.[3] For the co-sensitized NiO film with the E2 dye and catalyst **[1]** (**[1]**|E2|NiO), the E2 absorbance at 500 nm dominates his absorption spectrum and the presence of **[1]** was confirmed by FTIR (Fig. 1 in the main manuscript).

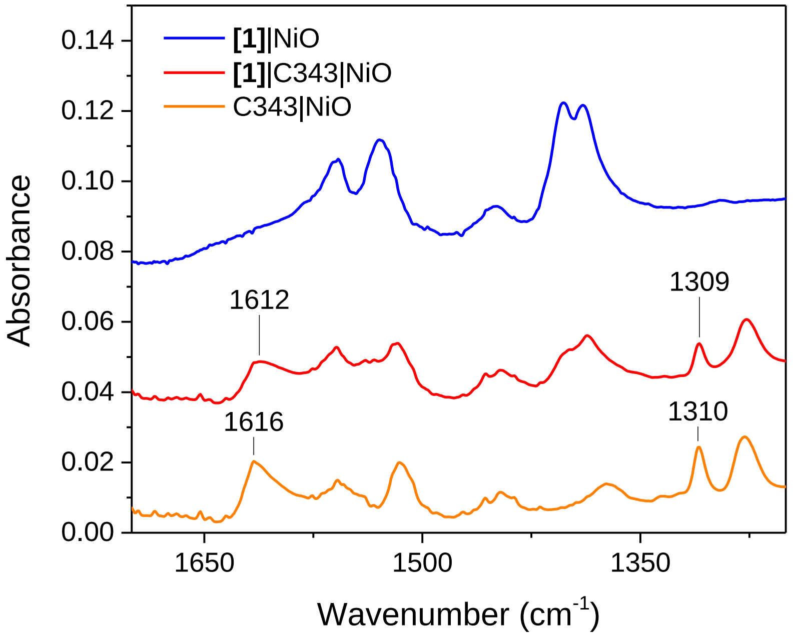

Fig. S2 IR absorption spectra of the co-sensitized **[1]**|C343|NiO (red), the reference **[1]**|NiO (blue) and C343|NiO (orange) in the region 1700 - 1200 cm^{-1}. The spectra have been offset by 0.04 absorbance units.

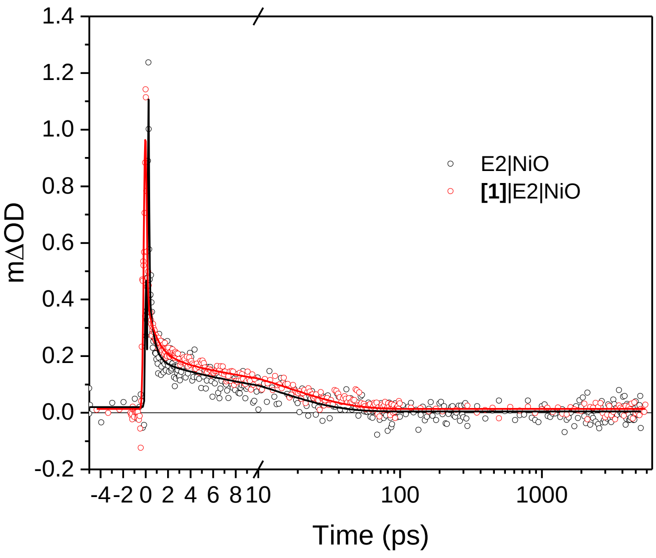

Fig. S3 Time evolution of the transient absorption at 2280 cm^{-1} for the E2-sensitized NiO film denoted E2|NiO (black dots) and for the NiO film co-sensitized with E2 and **[1]** denoted **[1]**|E2|NiO (red dots). The lines represent multi-exponential fitting to the data. For E2|NiO, the data could be represented by a tri-exponential expression with time constants 0.4 ps (64%), 14.0 ps (33%) and > 5 ns (3%). For **[1]**|E2|NiO, a bi-exponential expression with time constants 0.9 ps (54%) and 16.9 ps (46%) described well the data.

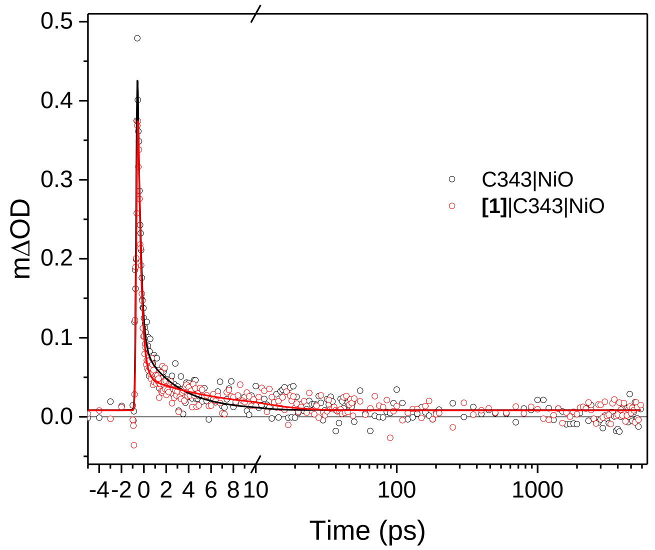

Fig. S4 Time evolution of the transient absorption at 2130 cm^{-1} for the C343-sensitized NiO film denoted C343|NiO (black dots) and for the NiO film co-sensitized with C343 and **[1]** denoted **[1]**|C343|NiO (red dots). The lines represent bi-exponential fits to the data. For C343|NiO, a bi-exponential fit gives the time constants and corresponding amplitudes: τ_1=0.2 ps (82%) and τ_2=3.2 ps (82%). For **[1]**|C343|NiO, a bi-exponential fit with time constants and corresponding amplitudes, τ_1=0.3 ps (92%) and τ_2=7.3 ps (8%), describes well the data.

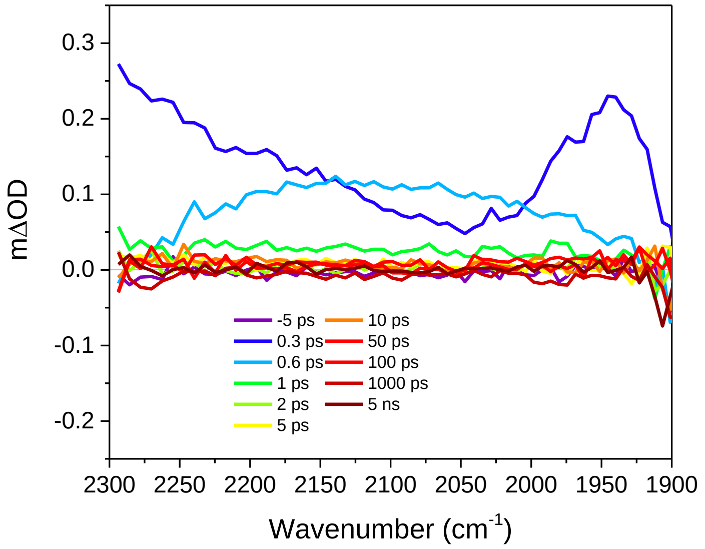

Fig. S5 Infrared TA spectra of the **[1]**|NiO reference film in propylene carbonate after excitation at 532 nm (400 nJ/pulse). The TA signal at early times (i.e. 0.3 ps and 0.6 ps after excitation) is an artifact that may in part arise from cross-phase modulation.

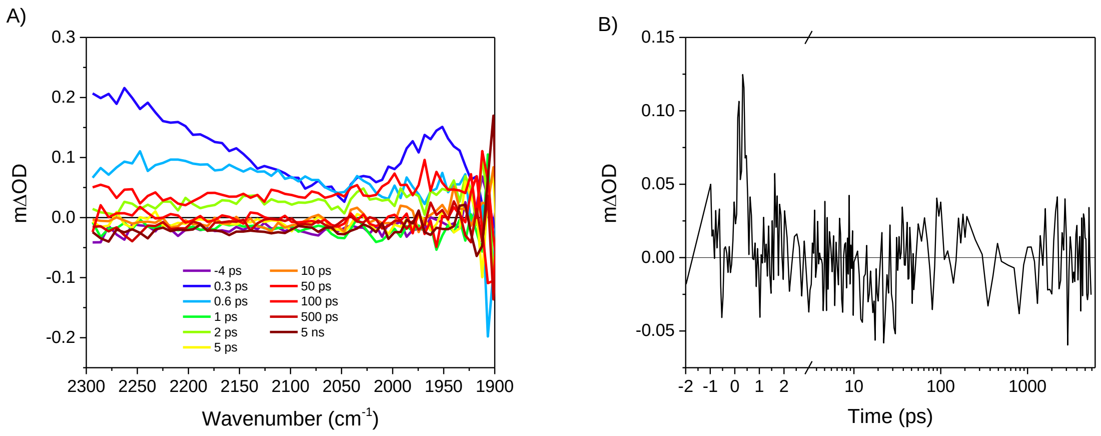

Fig. S6 A) Infrared TA spectra of the **[1]**|NiO reference in propylene carbonate after excitation at 440 nm (530 nJ/pulse) and B) Time evolution of the transient absorption at 2016 cm^{-1} for **[1]**|NiO. As in Fig. S6, the TA signal at early times (i.e. 0.3 ps and 0.6 ps after excitation) is an artifact that may in part arise from cross-phase modulation.

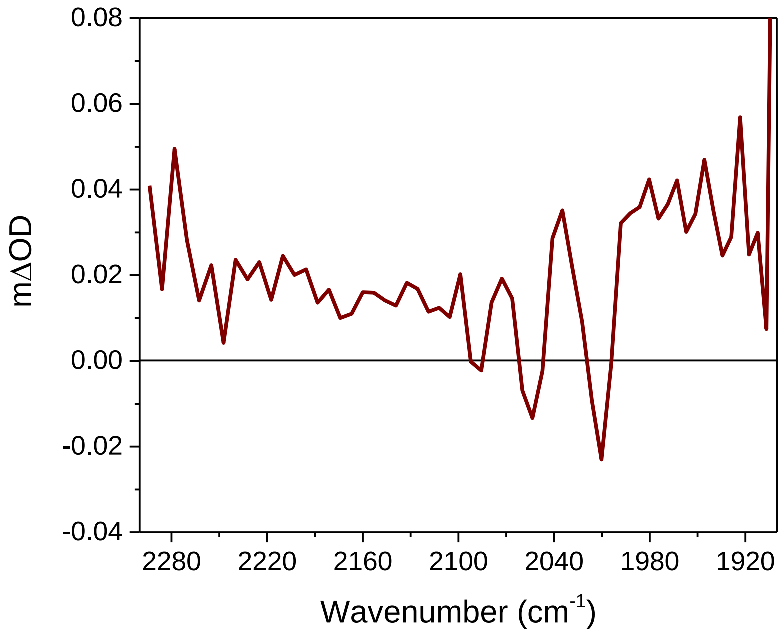

Fig. S7 Infrared TA spectrum of the **[1]**|C343|NiO co-sensitized film in propylene carbonate 5 ns after excitation at 440 nm (530 nJ/pulse).

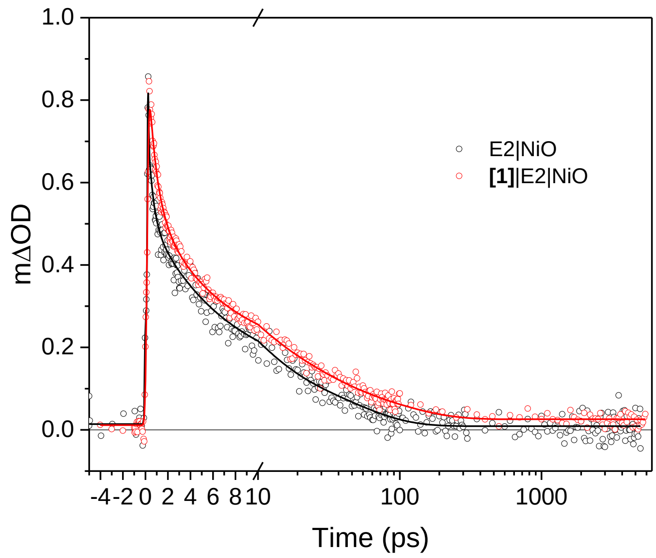

Fig. S8 Time evolution of the transient absorption at 2180 cm^{-1} for the E2-sensitized NiO film denoted E2|NiO (black dots) and for the NiO film co-sensitized with E2 and **[1]** denoted **[1]**|E2|NiO (red dots). The lines represent multi-exponential fits to the data. The corresponding time constants and corresponding are given below in Table S1.

Table S1 Fitting parameters for the data in Fig. S8

Sample	τ_1 (A1)	τ_2 (A2)	τ_3 (A3)	τ_4 (A4)
E2|NiO	0.5 ± 0.2 ps (34%)	6.3 ± 1.4 ps (44%)	42 ± 11 ps (22%)	> 5 ns (>1%)
**[1]**|E2|NiO	0.6 ± 0.1 ps (38%)	5.6 ± 0.1 ps (39%)	54 ± 4 ps (20%)	> 5 ns (2%)

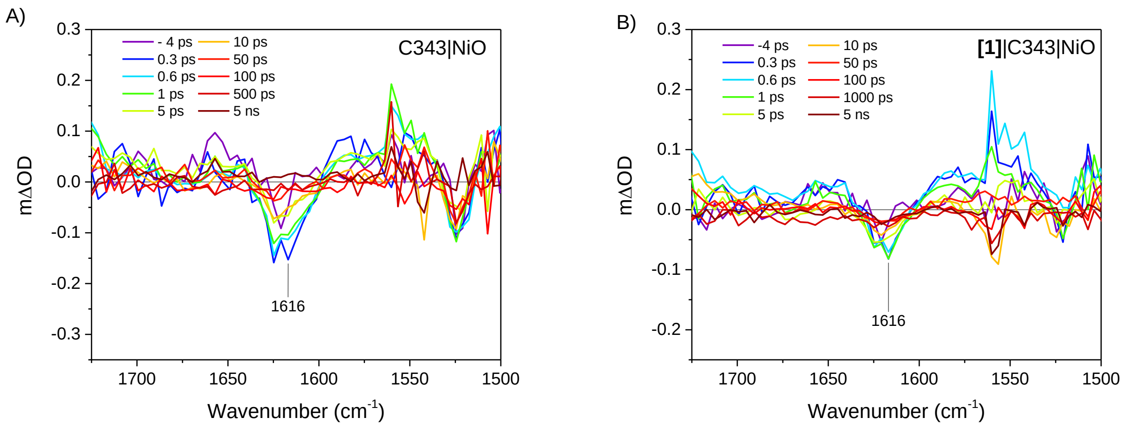

Fig. S9 Infrared TA spectra of A) the C343|NiO reference film and B) the **[1]**|C343|NiO co-sensitized NiO film in propylene carbonate after excitation at 440 nm (530 nJ/pulse).

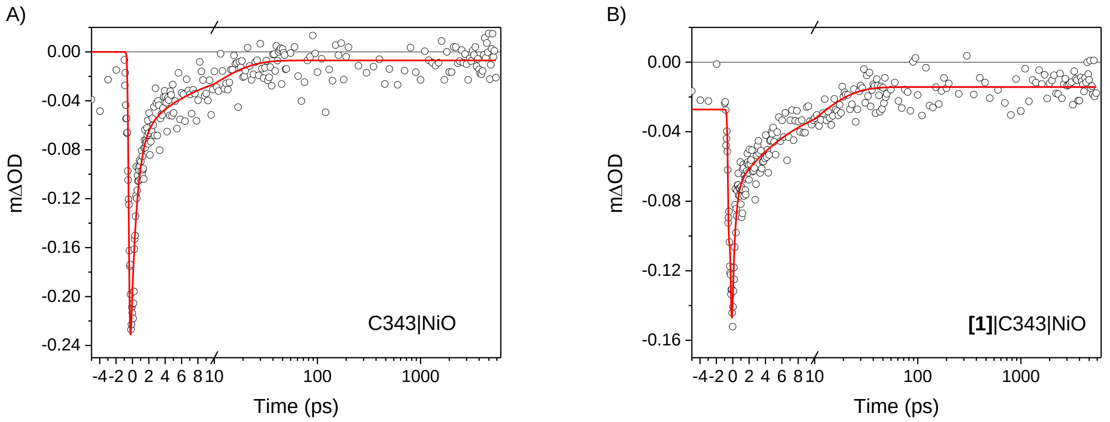

Fig. S10 Time evolution of the transient absorption at 1616 cm^{-1} for A) the C343|NiO reference film (dots) and B) for the **[1]**|C343|NiO co-sensitized film (dots). The red lines represent multi-exponential fits to the data. The corresponding time constants and corresponding are given below in Table S2. For **[1]**|C343|NiO, the bleach seems to recover on the same time scale as for C343|NiO. This might be explained by the poor experimental resolution in this wavenumber region, i.e. the CO vibration of the C343 dye and the NH stretch of **[1]** appear to be not well resolved. In comparison, the experimental resolution in the carbonyl region is much better.

Table S2 Fitting parameters for the data in Fig. S10

Sample	τ_1 (A1)	τ_2 (A2)	τ_3 (A3)
C343|NiO	0.7 ± 0.2 ps (-76%)	8.3 ± 3.4 ps (-22%)	> 5 ns (2%)
**[1]**|C343|NiO	0.3 ± 0.1 ps (-71%)	8.5 ± 1.6 ps (-24%)	> 5 ns (5%)

Further insight into for the mechanism of reduction of **[1]** in **[1]**|E2|NiO was obtained from ZrO_2 films (non-injecting material) co-sensitized with E2 and **[1]** in a reasonably comparable dye to catalyst ratio of ca. 1:2. The UV-vis and FTIR spectra of the co-sensitized film **[1]**|E2|ZrO_2 and reference films **[1]**|ZrO_2 and E2|ZrO_2 are shown in the supporting information (Fig. S11). Much to our surprise, the fs IR transient absorption spectra of **[1]**|E2|ZrO_2 are similar to the one observed for **[1]**|E2|NiO. After excitation of E2, immediate signal of reduced **[1]**$^-$ was observed in addition to the spectral features of the excited E2* for **[1]**|E2|ZrO_2 (Fig. S12). The reduced **[1]**$^-$ recombines on a time scale that is similar to the one observed for co-sensitized **[1]**|E2|NiO films (Table S3). A control experiment with **[1]** bound alone to ZrO_2 (**[1]**|ZrO_2) shows only featureless signals (Fig. S15), proving that E2 is necessary for the reduction of **[1]**. Such a similar behaviour in **[1]**|E2|ZrO_2 and **[1]**|E2|NiO suggest an identical mechanism for reduction of **[1]**, that is a direct electron transfer from the excited E2* to **[1]**. ZrO_2 band energies do not allow for either electron or hole injection from the excited dye. However, looking at the energetics, the excited E2* has indeed enough reducing power to reduce **[1]**, i.e. ΔG_0 = -0.53 eV (Fig. 1).[1]

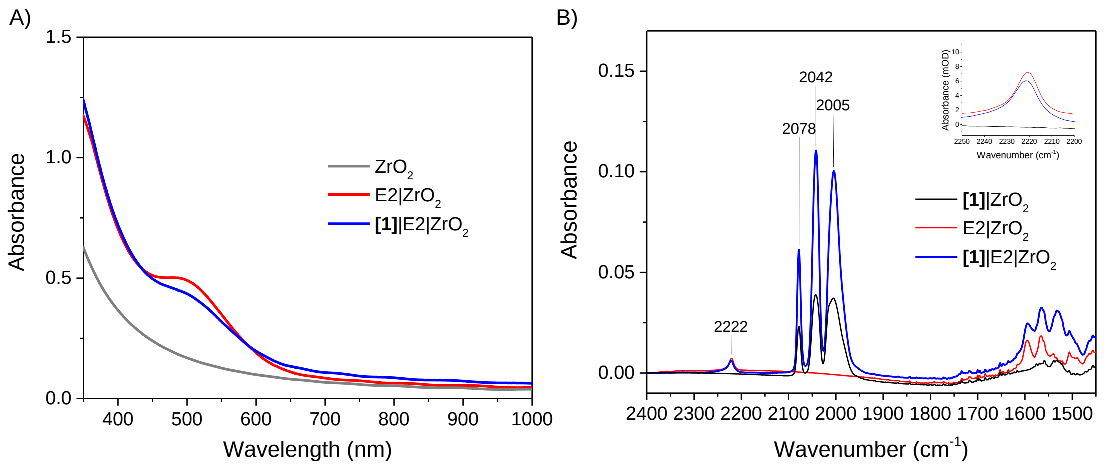

Fig. S11 A) UV-vis ground-state absorption spectra of the **[1]**|E2|ZrO_2 co-sensitized film (blue), E2|ZrO_2 reference film (red) and a ZrO_2 film before sensitization (gray). B) FTIR absorption spectra of the **[1]**|E2|ZrO_2 co-sensitized film (blue), the E2|ZrO_2 reference film (red) and the **[1]**|ZrO_2 reference film. Inset in panel B shows the region of the $\nu_{C\equiv N}$ stretch.

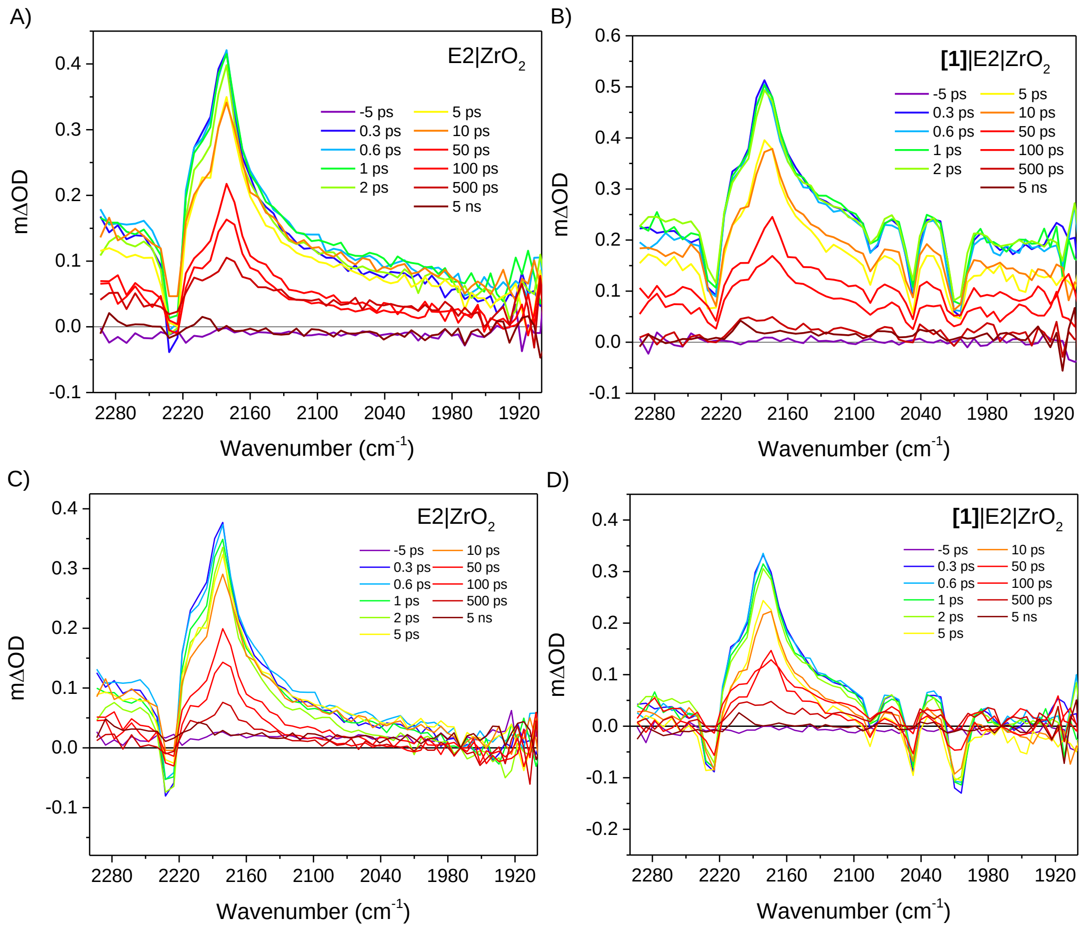

Fig. S12 (top right panel) Infrared TA spectra showing the reduction of the catalyst **[1]** via the carbonyl bands in the co-sensitized **[1]**|E2|ZrO_2. (top left panel) Infrared TA spectra of the sensitized E2|ZrO2 without catalyst. (bottom right panel) Infrared TA spectra after background correction at 1970 cm^{-1} showing the reduction of the catalyst **[1]** via the carbonyl bands in the co-sensitized **[1]**|E2|ZrO_2.(bottom left panel) Infrared TA spectra of the sensitized E2|ZrO2 without catalyst after background correction at 1970 cm^{-1}. The solvent was propylene carbonate. All the films were excited at 532 nm with 100 nJ pulse intensity.

Much to our surprise, the fs TA IR spectra after dye excitation at 532 nm clearly shows the characteristic peaks of the reduced catalyst, i.e. ground-state bleaches at 2086, 2047 and 2004 cm^{-1} and positive peaks at 2066, 2028 and 1986 cm^{-1} (Fig. S12B). In the cyano region, a ground-state bleaches at 2232 cm^{-1} and a positive band at 2180 cm^{-1} with a shoulder at 2210 cm^{-1} are observed for both E2|ZrO_2 and **[1]**|E2|ZrO_2. No significant differences in shape and position are observed between the samples.

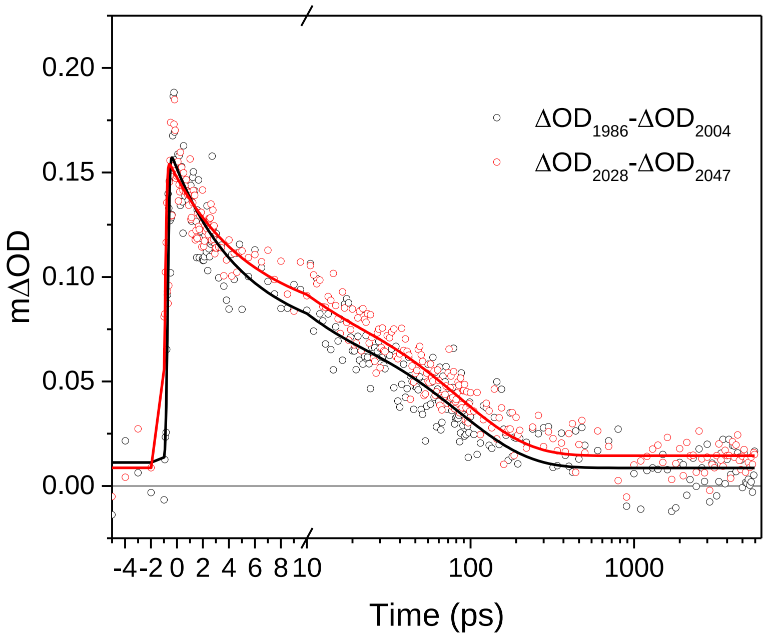

Fig. S13 Kinetics of reduction of **[1]** upon photoexcitation of the co-sensitized dye in a **[1]**|E2|ZrO2 film. In red, the kinetics of reduction of **[1]** is obtained from the difference between the transient absorption signals of **[1]**$^-$ and **[1]** taken at 2028 cm^{-1} and 2047 cm^{-1}, respectively. In black, the kinetics of reduction of **[1]** is obtained from the difference between the transient absorption signals of **[1]**$^-$ and **[1]** taken at 1986 cm^{-1} and 2004 cm^{-1}, respectively. Both traces are within experimental error the same. The solid lines are fits with three exponentials to the experimental data (Table S3).

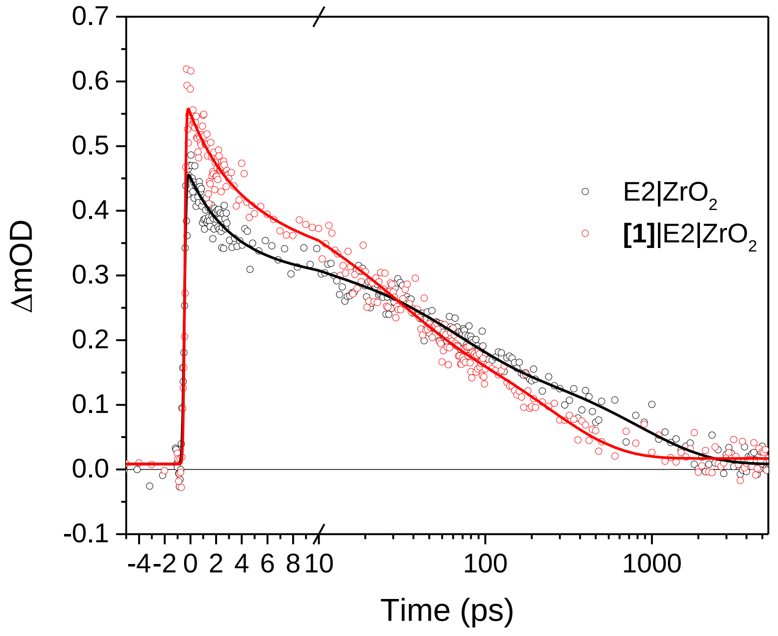

Fig. S14 Time evolution of the transient absorption at 2188 cm^{-1} for the **[1]**|E2|ZrO_2 co-sensitized film (red dots) and the E2|ZrO_2 film (black dots). The lines represent multi-exponential fits to the data. The corresponding time constants and corresponding are given below in Table S3.

Table S3 Fitting parameters for the data in Fig. S12 and Fig. S14

Sample	Probe wavenumber	τ_1 (A1)	τ_2 (A2)	τ_3 (A3)	τ_4 (A4)
E2|ZrO_2	2188 cm^{-1}	3.1 ± 0.4 ps (30%)	59 ± 8 ps (35%)	821 ± 125 ps (35%)	> 5 ns (>1%)
**[1]**|E2|ZrO_2	2184 cm^{-1}	3 ± ps (26%)	24 ± 3 ps (35%)	238 ± 27 ps (37%)	> 5 ns (1%)
**[1]**|E2|ZrO_2	1986-2004 cm^{-1}	4.9 ± 0.5 ps (52%)	85 ± 7 ps (47%)	> 5 ns (1%)	
**[1]**|E2|ZrO_2	2028-2047 cm^{-1}	5.4 ± 0.6 ps (44%)	84± 6 ps (52%)	> 5 ns (4%)	

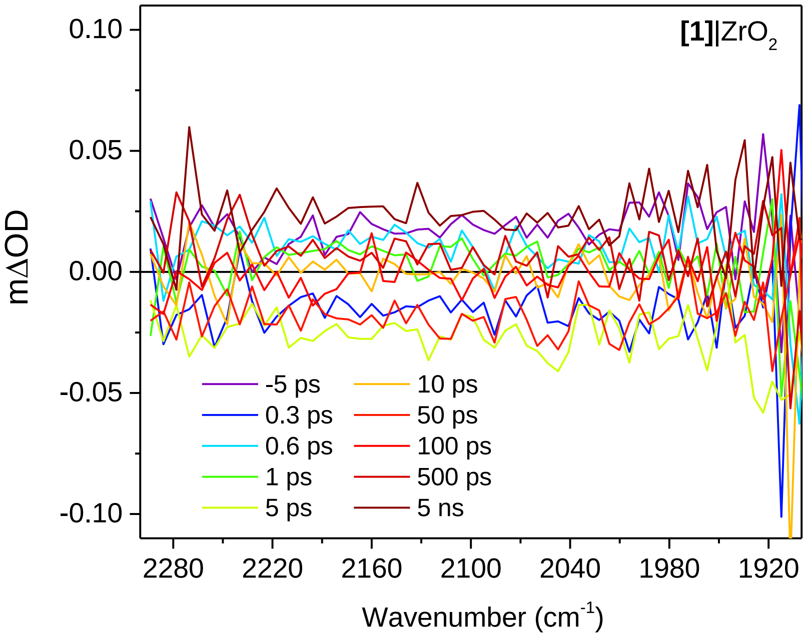

Fig. S15 Infrared TA spectra of the **[1]**|ZrO_2 reference film with propylene carbonate as solvent after excitation at 532 nm (200 nJ/pulse).

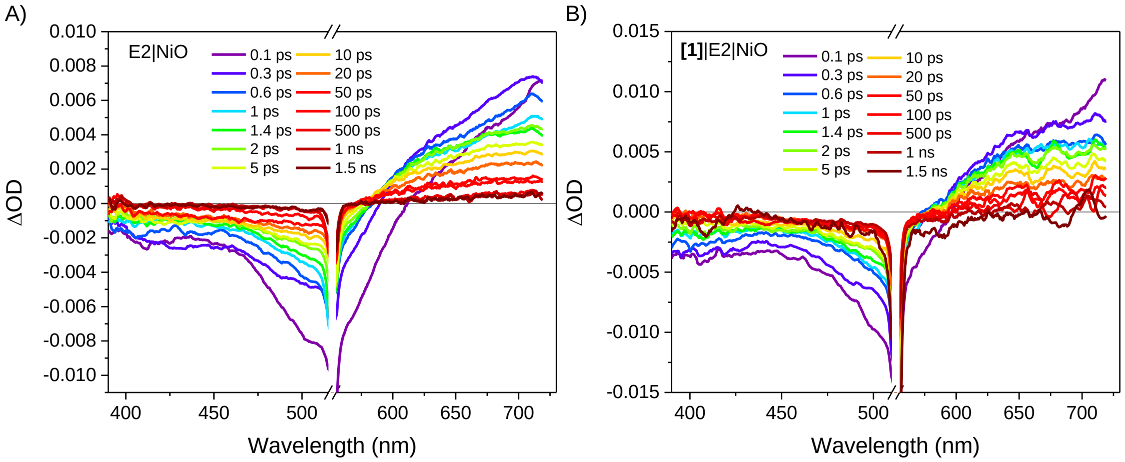

Fig. S16 UV-Vis TA spectra of A) the E2|NiO film and B) the co-sensitized **[1]**|E2|NiO film after excitation at 532 nm (nJ/pulse). The solvent was propylene carbonate

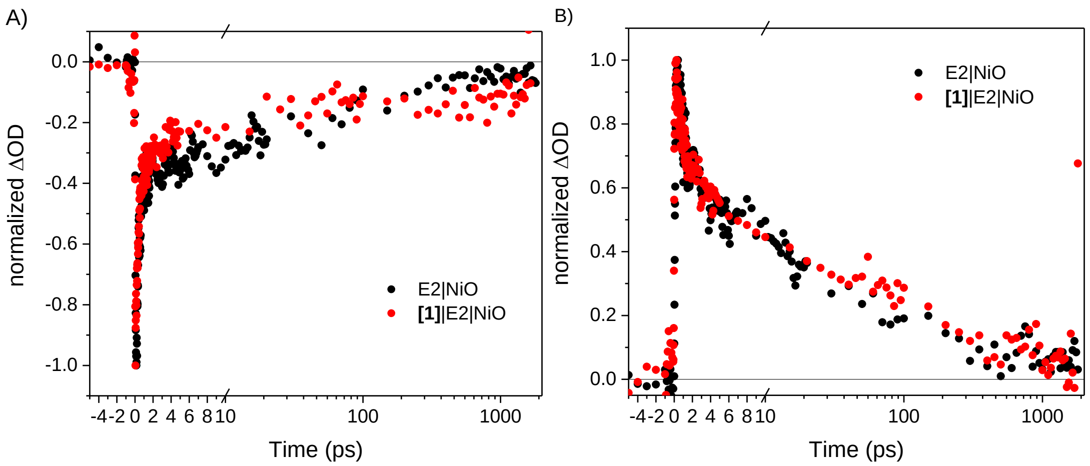

Fig. S17 Normalized time evolution of the visible transient absorption at A) 470 nm and B) at 650 nm for the **[1]**|E2|NiO co-sensitized film (red dots) and the E2|NiO film (black dots).

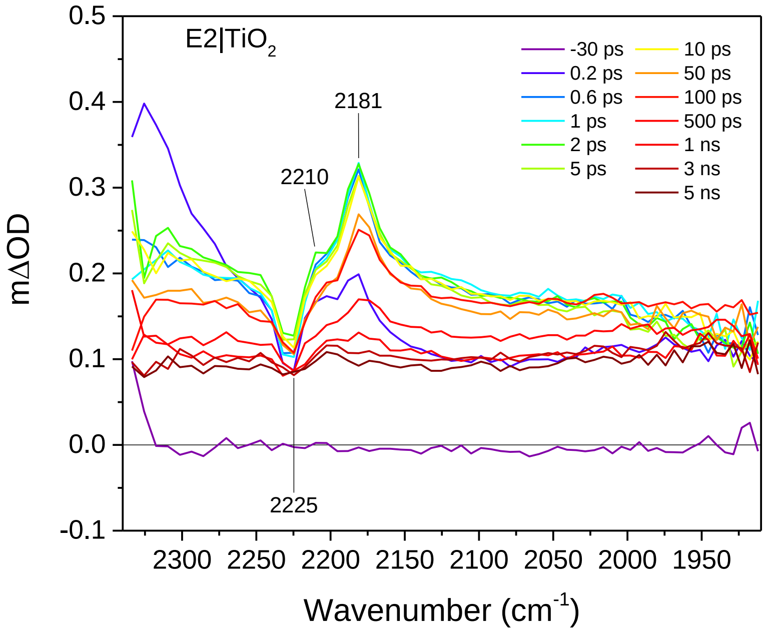

Fig. S18 Infrared TA spectra of the E2|TiO_2 film with propylene carbonate as solvent after excitation at 532 nm (333 nJ/pulse).

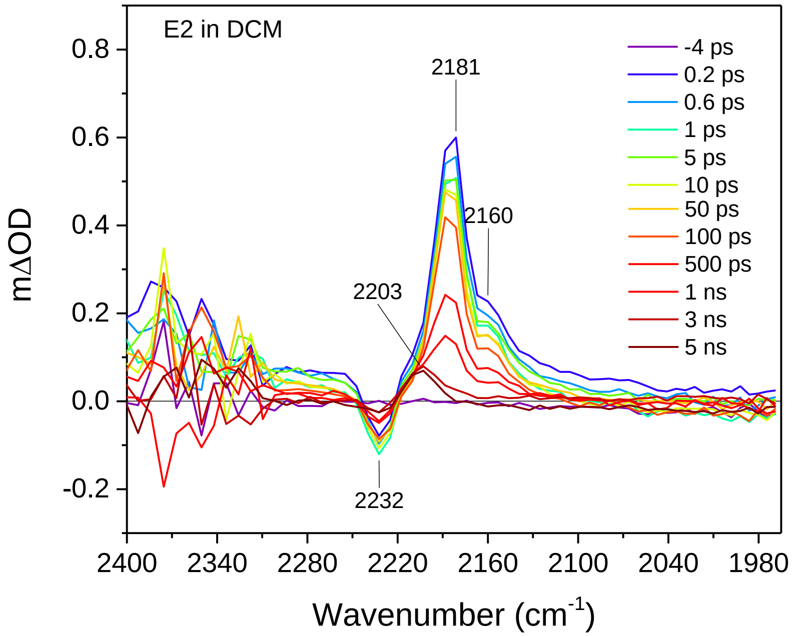

Fig. S19 Infrared TA spectra of the dye E2 in dichloromethane after excitation at 532 nm (333 nJ/pulse).

References

1. E. Sheibani, L. Zhang, P. Liu, B. Xu, E. Mijangos, G. Boschloo, A. Hagfeldt, L. Hammarstrom, L. Kloo and H. Tian, *RSC Advances*, 2016, **6**, 18165-18177.
2. L. J. Antila, P. Ghamgosar, S. Maji, H. Tian, S. Ott and L. Hammarström, *ACS Energy Letters*, 2016, **1**, 1106-1111.
3. M. Mirmohades, S. Pullen, M. Stein, S. Maji, S. Ott, L. Hammarström and R. Lomoth, *Journal of the American Chemical Society*, 2014, **136**, 17366-17369.
